# Supplementary material for: Transmembrane Helices 7 and 8 Confer Aggregation Sensitivity to the Cystic Fibrosis Transmembrane Conductance Regulator
Source: Int J Mol Sci. 2023 Oct 30;24(21):15741. doi: 10.3390/ijms242115741 (PMC10648718; doi:10.3390/ijms242115741)
Supplement: Supplementary file 1 [file ijms-24-15741-s001.zip › ijms-2619896-supplementary.pdf]

Table S1. Primers used for generation of constructs used in this study

Construct Name	Primer Sequence (5’ to 3’)
L998X	F: TCCTGAACCTGATGA CACACTC<br>R: CCCTCGAGCTACAATTGGATGAA GTCAAATATGGTAAG
D1202X	F: TCCTGAACCTGATGA CACACTC<br>R: CCCTCGAGC TAATCTTTCTTCACGTGTGAATTCTC
Y1219X	F: TCCTGAACCTGATGACACACTC<br>R: CCGCTCGAGCTATTTTGCTGTGAGATCTTTGAC

Table S2. Antibodies used in this study

Antibody	Domain	Epitope	Source
E1-22	TMD1	107-118	This lab
G449	R	645-835	A. Nairn
TMD2C	TMD2	364-381	This lab
MrPink	NBD1	multiple in NBD1	This lab
MrBrown	HA	YPYDVPDYA	This lab
